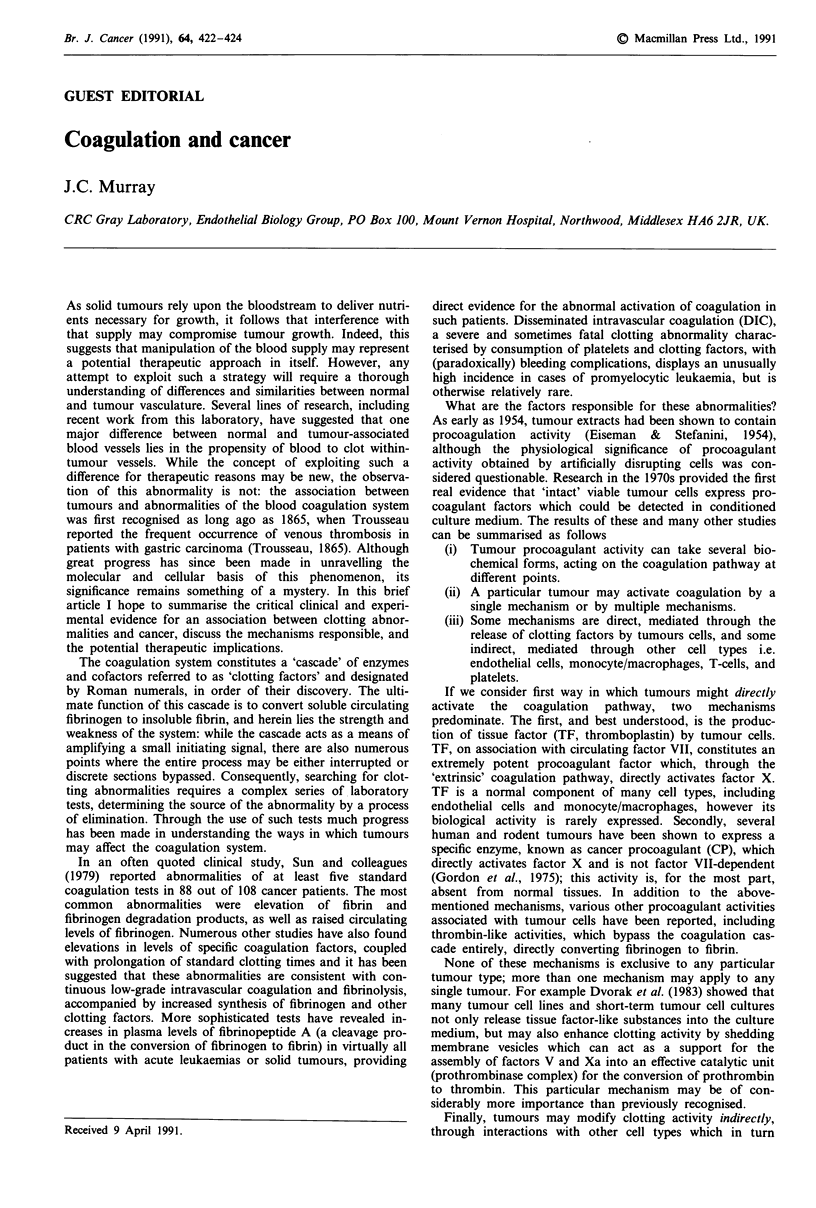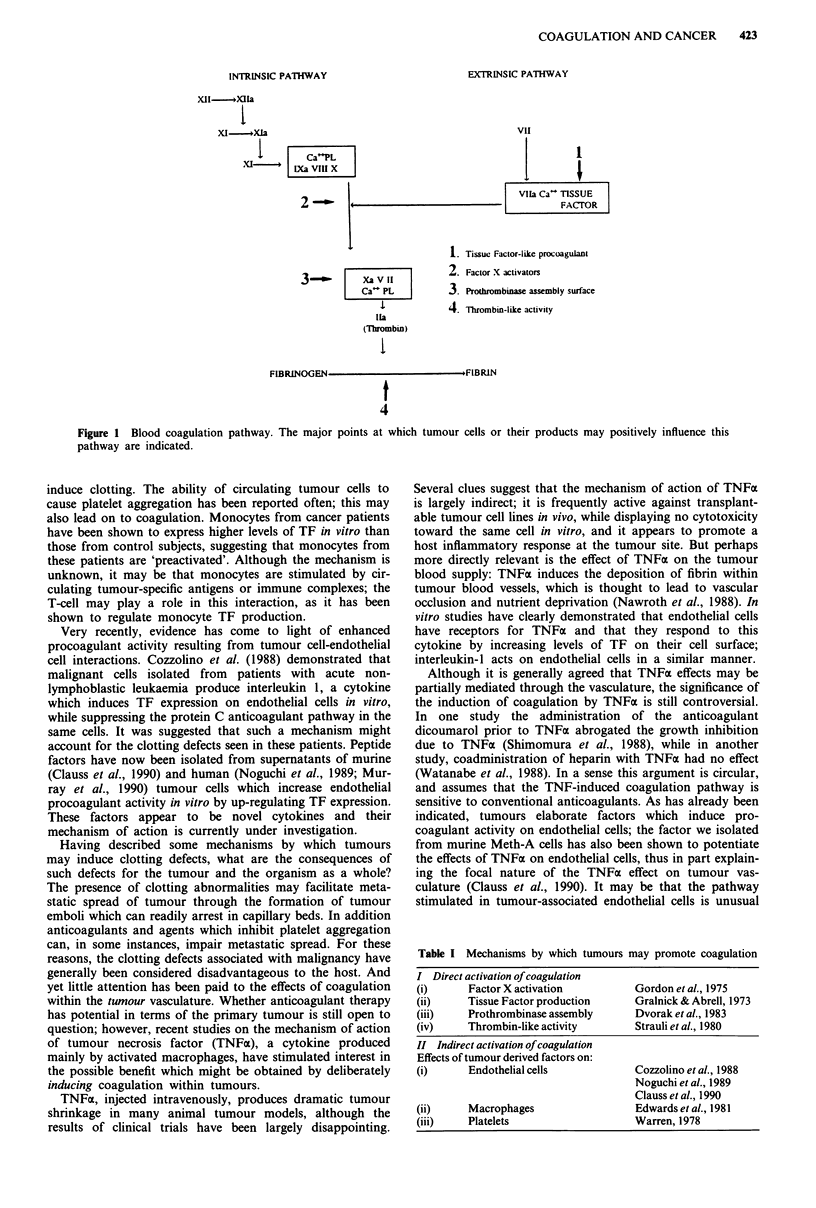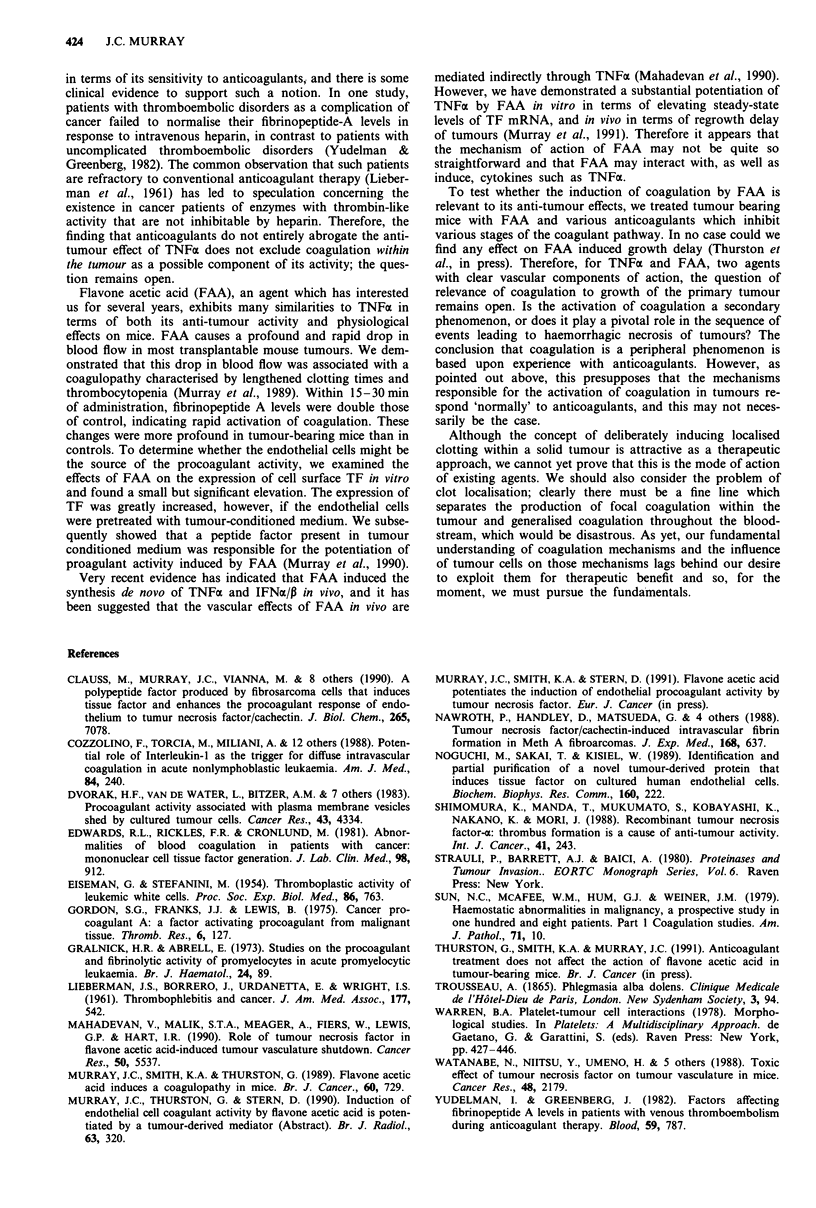# Coagulation and cancer.

**DOI:** 10.1038/bjc.1991.325

**Published:** 1991-09

**Authors:** J. C. Murray


Br. J. Cancer (1991), 64, 422-424 © Macmillan Press Ltd., 1991

GUEST EDITORIAL

### Coagulation and cancer

J.C. Murray

*CRC Gray Laboratory, Endothelial Biology Group, PO Box 100, Mount Vernon Hospital, Northwood, Middlesex HA6 2JR, UK.*

As solid tumours rely upon the bloodstream to deliver nutrients necessary for growth, it follows that interference with that supply may compromise tumour growth. Indeed, this suggests that manipulation of the blood supply may represent a potential therapeutic approach in itself. However, any attempt to exploit such a strategy will require a thorough understanding of differences and similarities between normal and tumour vasculature. Several lines of research, including recent work from this laboratory, have suggested that one major difference between normal and tumour-associated blood vessels lies in the propensity of blood to clot within-tumour vessels. While the concept of exploiting such a difference for therapeutic reasons may be new, the observation of this abnormality is not: the association between tumours and abnormalities of the blood coagulation system was first recognised as long ago as 1865, when Trousseau reported the frequent occurrence of venous thrombosis in patients with gastric carcinoma (Trousseau, 1865). Although great progress has since been made in unravelling the molecular and cellular basis of this phenomenon, its significance remains something of a mystery. In this brief article I hope to summarise the critical clinical and experimental evidence for an association between clotting abnormalities and cancer, discuss the mechanisms responsible, and the potential therapeutic implications.

The coagulation system constitutes a 'cascade' of enzymes and cofactors referred to as 'clotting factors' and designated by Roman numerals, in order of their discovery. The ultimate function of this cascade is to convert soluble circulating fibrinogen to insoluble fibrin, and herein lies the strength and weakness of the system: while the cascade acts as a means of amplifying a small initiating signal, there are also numerous points where the entire process may be either interrupted or discrete sections bypassed. Consequently, searching for clotting abnormalities requires a complex series of laboratory tests, determining the source of the abnormality by a process of elimination. Through the use of such tests much progress has been made in understanding the ways in which tumours may affect the coagulation system.

In an often quoted clinical study, Sun and colleagues (1979) reported abnormalities of at least five standard coagulation tests in 88 out of 108 cancer patients. The most common abnormalities were elevation of fibrin and fibrinogen degradation products, as well as raised circulating levels of fibrinogen. Numerous other studies have also found elevations in levels of specific coagulation factors, coupled with prolongation of standard clotting times and it has been suggested that these abnormalities are consistent with continuous low-grade intravascular coagulation and fibrinolysis, accompanied by increased synthesis of fibrinogen and other clotting factors. More sophisticated tests have revealed increases in plasma levels of fibrinopeptide A (a cleavage product in the conversion of fibrinogen to fibrin) in virtually all patients with acute leukaemias or solid tumours, providing direct evidence for the abnormal activation of coagulation in such patients. Disseminated intravascular coagulation (DIC), a severe and sometimes fatal clotting abnormality characterised by consumption of platelets and clotting factors, with (paradoxically) bleeding complications, displays an unusually high incidence in cases of promyelocytic leukaemia, but is otherwise relatively rare.

What are the factors responsible for these abnormalities? As early as 1954, tumour extracts had been shown to contain procoagulation activity (Eiseman & Stefanini, 1954), although the physiological significance of procoagulant activity obtained by artificially disrupting cells was considered questionable. Research in the 1970s provided the first real evidence that 'intact' viable tumour cells express procoagulant factors which could be detected in conditioned culture medium. The results of these and many other studies can be summarised as follows

(i) Tumour procoagulant activity can take several biochemical forms, acting on the coagulation pathway at different points.
(ii) A particular tumour may activate coagulation by a single mechanism or by multiple mechanisms.
(iii) Some mechanisms are direct, mediated through the release of clotting factors by tumours cells, and some indirect, mediated through other cell types i.e. endothelial cells, monocyte/macrophages, T-cells, and platelets.

If we consider first way in which tumours might *directly* activate the coagulation pathway, two mechanisms predominate. The first, and best understood, is the production of tissue factor (TF, thromboplastin) by tumour cells. TF, on association with circulating factor VII, constitutes an extremely potent procoagulant factor which, through the 'extrinsic' coagulation pathway, directly activates factor X. TF is a normal component of many cell types, including endothelial cells and monocyte/macrophages, however its biological activity is rarely expressed. Secondly, several human and rodent tumours have been shown to express a specific enzyme, known as cancer procoagulant (CP), which directly activates factor X and is not factor VII-dependent (Gordon *et al.*, 1975); this activity is, for the most part, absent from normal tissues. In addition to the above-mentioned mechanisms, various other procoagulant activities associated with tumour cells have been reported, including thrombin-like activities, which bypass the coagulation cascade entirely, directly converting fibrinogen to fibrin.

None of these mechanisms is exclusive to any particular tumour type; more than one mechanism may apply to any single tumour. For example Dvorak *et al.* (1983) showed that many tumour cell lines and short-term tumour cell cultures not only release tissue factor-like substances into the culture medium, but may also enhance clotting activity by shedding membrane vesicles which can act as a support for the assembly of factors V and Xa into an effective catalytic unit (prothrombinase complex) for the conversion of prothrombin to thrombin. This particular mechanism may be of considerably more importance than previously recognised.

Finally, tumours may modify clotting activity *indirectly*, through interactions with other cell types which in turn

Received 9 April 1991.

**Figure 1** Blood coagulation pathway. The major points at which tumour cells or their products may positively influence this pathway are indicated.

induce clotting. The ability of circulating tumour cells to cause platelet aggregation has been reported often; this may also lead on to coagulation. Monocytes from cancer patients have been shown to express higher levels of TF *in vitro* than those from control subjects, suggesting that monocytes from these patients are 'preactivated'. Although the mechanism is unknown, it may be that monocytes are stimulated by circulating tumour-specific antigens or immune complexes; the T-cell may play a role in this interaction, as it has been shown to regulate monocyte TF production.

Very recently, evidence has come to light of enhanced procoagulant activity resulting from tumour cell-endothelial cell interactions. Cozzolino *et al.* (1988) demonstrated that malignant cells isolated from patients with acute non-lymphoblastic leukaemia produce interleukin 1, a cytokine which induces TF expression on endothelial cells *in vitro*, while suppressing the protein C anticoagulant pathway in the same cells. It was suggested that such a mechanism might account for the clotting defects seen in these patients. Peptide factors have now been isolated from supernatants of murine (Clauss *et al.*, 1990) and human (Noguchi *et al.*, 1989; Murray *et al.*, 1990) tumour cells which increase endothelial procoagulant activity *in vitro* by up-regulating TF expression. These factors appear to be novel cytokines and their mechanism of action is currently under investigation.

Having described some mechanisms by which tumours may induce clotting defects, what are the consequences of such defects for the tumour and the organism as a whole? The presence of clotting abnormalities may facilitate metastatic spread of tumour through the formation of tumour emboli which can readily arrest in capillary beds. In addition anticoagulants and agents which inhibit platelet aggregation can, in some instances, impair metastatic spread. For these reasons, the clotting defects associated with malignancy have generally been considered disadvantageous to the host. And yet little attention has been paid to the effects of coagulation within the *tumour* vasculature. Whether anticoagulant therapy has potential in terms of the primary tumour is still open to question; however, recent studies on the mechanism of action of tumour necrosis factor (TNFα), a cytokine produced mainly by activated macrophages, have stimulated interest in the possible benefit which might be obtained by deliberately *inducing* coagulation within tumours.

TNFα, injected intravenously, produces dramatic tumour shrinkage in many animal tumour models, although the results of clinical trials have been largely disappointing. Several clues suggest that the mechanism of action of TNFα is largely indirect; it is frequently active against transplantable tumour cell lines *in vivo*, while displaying no cytotoxicity toward the same cell *in vitro*, and it appears to promote a host inflammatory response at the tumour site. But perhaps more directly relevant is the effect of TNFα on the tumour blood supply: TNFα induces the deposition of fibrin within tumour blood vessels, which is thought to lead to vascular occlusion and nutrient deprivation (Nawroth *et al.*, 1988). *In vitro* studies have clearly demonstrated that endothelial cells have receptors for TNFα and that they respond to this cytokine by increasing levels of TF on their cell surface; interleukin-1 acts on endothelial cells in a similar manner.

Although it is generally agreed that TNFα effects may be partially mediated through the vasculature, the significance of the induction of coagulation by TNFα is still controversial. In one study the administration of the anticoagulant dicoumarol prior to TNFα abrogated the growth inhibition due to TNFα (Shimomura *et al.*, 1988), while in another study, coadministration of heparin with TNFα had no effect (Watanabe *et al.*, 1988). In a sense this argument is circular, and assumes that the TNF-induced coagulation pathway is sensitive to conventional anticoagulants. As has already been indicated, tumours elaborate factors which induce procoagulant activity on endothelial cells; the factor we isolated from murine Meth-A cells has also been shown to potentiate the effects of TNFα on endothelial cells, thus in part explaining the focal nature of the TNFα effect on tumour vasculature (Clauss *et al.*, 1990). It may be that the pathway stimulated in tumour-associated endothelial cells is unusual

**Table I** Mechanisms by which tumours may promote coagulation

| | | |
|---|---|---|
| *I Direct activation of coagulation* | | |
| (i) | Factor X activation | Gordon *et al.*, 1975 |
| (ii) | Tissue Factor production | Gralnick & Abrell, 1973 |
| (iii) | Prothrombinase assembly | Dvorak *et al.*, 1983 |
| (iv) | Thrombin-like activity | Strauli *et al.*, 1980 |
| *II Indirect activation of coagulation* | | |
| Effects of tumour derived factors on: | | |
| (i) | Endothelial cells | Cozzolino *et al.*, 1988<br>Noguchi *et al.*, 1989<br>Clauss *et al.*, 1990 |
| (ii) | Macrophages | Edwards *et al.*, 1981 |
| (iii) | Platelets | Warren, 1978 |

in terms of its sensitivity to anticoagulants, and there is some clinical evidence to support such a notion. In one study, patients with thromboembolic disorders as a complication of cancer failed to normalise their fibrinopeptide-A levels in response to intravenous heparin, in contrast to patients with uncomplicated thromboembolic disorders (Yudelman & Greenberg, 1982). The common observation that such patients are refractory to conventional anticoagulant therapy (Lieberman *et al.*, 1961) has led to speculation concerning the existence in cancer patients of enzymes with thrombin-like activity that are not inhibitable by heparin. Therefore, the finding that anticoagulants do not entirely abrogate the anti-tumour effect of TNFα does not exclude coagulation *within the tumour* as a possible component of its activity; the question remains open.

Flavone acetic acid (FAA), an agent which has interested us for several years, exhibits many similarities to TNFα in terms of both its anti-tumour activity and physiological effects on mice. FAA causes a profound and rapid drop in blood flow in most transplantable mouse tumours. We demonstrated that this drop in blood flow was associated with a coagulopathy characterised by lengthened clotting times and thrombocytopenia (Murray *et al.*, 1989). Within 15–30 min of administration, fibrinopeptide A levels were double those of control, indicating rapid activation of coagulation. These changes were more profound in tumour-bearing mice than in controls. To determine whether the endothelial cells might be the source of the procoagulant activity, we examined the effects of FAA on the expression of cell surface TF *in vitro* and found a small but significant elevation. The expression of TF was greatly increased, however, if the endothelial cells were pretreated with tumour-conditioned medium. We subsequently showed that a peptide factor present in tumour conditioned medium was responsible for the potentiation of proagulant activity induced by FAA (Murray *et al.*, 1990).

Very recent evidence has indicated that FAA induced the synthesis *de novo* of TNFα and IFNα/β *in vivo*, and it has been suggested that the vascular effects of FAA *in vivo* are mediated indirectly through TNFα (Mahadevan *et al.*, 1990). However, we have demonstrated a substantial potentiation of TNFα by FAA *in vitro* in terms of elevating steady-state levels of TF mRNA, and *in vivo* in terms of regrowth delay of tumours (Murray *et al.*, 1991). Therefore it appears that the mechanism of action of FAA may not be quite so straightforward and that FAA may interact with, as well as induce, cytokines such as TNFα.

To test whether the induction of coagulation by FAA is relevant to its anti-tumour effects, we treated tumour bearing mice with FAA and various anticoagulants which inhibit various stages of the coagulant pathway. In no case could we find any effect on FAA induced growth delay (Thurston *et al.*, in press). Therefore, for TNFα and FAA, two agents with clear vascular components of action, the question of relevance of coagulation to growth of the primary tumour remains open. Is the activation of coagulation a secondary phenomenon, or does it play a pivotal role in the sequence of events leading to haemorrhagic necrosis of tumours? The conclusion that coagulation is a peripheral phenomenon is based upon experience with anticoagulants. However, as pointed out above, this presupposes that the mechanisms responsible for the activation of coagulation in tumours respond 'normally' to anticoagulants, and this may not necessarily be the case.

Although the concept of deliberately inducing localised clotting within a solid tumour is attractive as a therapeutic approach, we cannot yet prove that this is the mode of action of existing agents. We should also consider the problem of clot localisation; clearly there must be a fine line which separates the production of focal coagulation within the tumour and generalised coagulation throughout the bloodstream, which would be disastrous. As yet, our fundamental understanding of coagulation mechanisms and the influence of tumour cells on those mechanisms lags behind our desire to exploit them for therapeutic benefit and so, for the moment, we must pursue the fundamentals.

### References

CLAUSS, M., MURRAY, J.C., VIANNA, M. & 8 others (1990). A polypeptide factor produced by fibrosarcoma cells that induces tissue factor and enhances the procoagulant response of endothelium to tumur necrosis factor/cachectin. *J. Biol. Chem.*, **265**, 7078.

COZZOLINO, F., TORCIA, M., MILIANI, A. & 12 others (1988). Potential role of Interleukin-1 as the trigger for diffuse intravascular coagulation in acute nonlymphoblastic leukaemia. *Am. J. Med.*, **84**, 240.

DVORAK, H.F., VAN DE WATER, L., BITZER, A.M. & 7 others (1983). Procoagulant activity associated with plasma membrane vesicles shed by cultured tumour cells. *Cancer Res.*, **43**, 4334.

EDWARDS, R.L., RICKLES, F.R. & CRONLUND, M. (1981). Abnormalities of blood coagulation in patients with cancer: mononuclear cell tissue factor generation. *J. Lab. Clin. Med.*, **98**, 912.

EISEMAN, G. & STEFANINI, M. (1954). Thromboplastic activity of leukemic white cells. *Proc. Soc. Exp. Biol. Med.*, **86**, 763.

GORDON, S.G., FRANKS, J.J. & LEWIS, B. (1975). Cancer procoagulant A: a factor activating procoagulant from malignant tissue. *Thromb. Res.*, **6**, 127.

GRALNICK, H.R. & ABRELL, E. (1973). Studies on the procoagulant and fibrinolytic activity of promyelocytes in acute promyelocytic leukaemia. *Br. J. Haematol.*, **24**, 89.

LIEBERMAN, J.S., BORRERO, J., URDANETTA, E. & WRIGHT, I.S. (1961). Thrombophlebitis and cancer. *J. Am. Med. Assoc.*, **177**, 542.

MAHADEVAN, V., MALIK, S.T.A., MEAGER, A., FIERS, W., LEWIS, G.P. & HART, I.R. (1990). Role of tumour necrosis factor in flavone acetic acid-induced tumour vasculature shutdown. *Cancer Res.*, **50**, 5537.

MURRAY, J.C., SMITH, K.A. & THURSTON, G. (1989). Flavone acetic acid induces a coagulopathy in mice. *Br. J. Cancer.*, **60**, 729.

MURRAY, J.C., THURSTON, G. & STERN, D. (1990). Induction of endothelial cell coagulant activity by flavone acetic acid is potentiated by a tumour-derived mediator (Abstract). *Br. J. Radiol.*, **63**, 320.

MURRAY, J.C., SMITH, K.A. & STERN, D. (1991). Flavone acetic acid potentiates the induction of endothelial procoagulant activity by tumour necrosis factor. *Eur. J. Cancer* (in press).

NAWROTH, P., HANDLEY, D., MATSUEDA, G. & 4 others (1988). Tumour necrosis factor/cachectin-induced intravascular fibrin formation in Meth A fibroarcomas. *J. Exp. Med.*, **168**, 637.

NOGUCHI, M., SAKAI, T. & KISIEL, W. (1989). Identification and partial purification of a novel tumour-derived protein that induces tissue factor on cultured human endothelial cells. *Biochem. Biophys. Res. Comm.*, **160**, 222.

SHIMOMURA, K., MANDA, T., MUKUMATO, S., KOBAYASHI, K., NAKANO, K. & MORI, J. (1988). Recombinant tumour necrosis factor-α: thrombus formation is a cause of anti-tumour activity. *Int. J. Cancer.*, **41**, 243.

STRAULI, P., BARRETT, A.J. & BAICI, A. (1980). *Proteinases and Tumour Invasion.. EORTC Monograph Series, Vol. 6.* Raven Press: New York.

SUN, N.C., MCAFEE, W.M., HUM, G.J. & WEINER, J.M. (1979). Haemostatic abnormalities in malignancy, a prospective study in one hundred and eight patients. Part 1 Coagulation studies. *Am. J. Pathol.*, **71**, 10.

THURSTON, G., SMITH, K.A. & MURRAY, J.C. (1991). Anticoagulant treatment does not affect the action of flavone acetic acid in tumour-bearing mice. *Br. J. Cancer* (in press).

TROUSSEAU, A. (1865). Phlegmasia alba dolens. *Clinique Medicale de l'Hôtel-Dieu de Paris, London. New Sydenham Society*, **3**, 94.

WARREN, B.A. Platelet-tumour cell interactions (1978). Morphological studies. In *Platelets: A Multidisciplinary Approach.* de Gaetano, G. & Garattini, S. (eds). Raven Press: New York, pp. 427–446.

WATANABE, N., NIITSU, Y., UMENO, H. & 5 others (1988). Toxic effect of tumour necrosis factor on tumour vasculature in mice. *Cancer Res.*, **48**, 2179.

YUDELMAN, I. & GREENBERG, J. (1982). Factors affecting fibrinopeptide A levels in patients with venous thromboembolism during anticoagulant therapy. *Blood*, **59**, 787.